# The role of DSC MR perfusion in predicting IDH mutation and 1p19q codeletion status in gliomas: meta-analysis and technical considerations

**DOI:** 10.1007/s00234-023-03154-5

**Published:** 2023-05-13

**Authors:** Loizos Siakallis, Constantin-Cristian Topriceanu, Jasmina Panovska-Griffiths, Sotirios Bisdas

**Affiliations:** 1 University College London (UCL) Queen Square Institute of Neurology, London, UK; 2 Lysholm Department of Neuroradiology, The National Hospital for Neurology and Neurosurgery, University College London Hospitals (UCLH) NHS Foundation Trust, London, UK; 3 UCL Institute of Cardiovascular Science, University College London, London, UK; 4 The Big Data Institute and the Pandemic Sciences Institute, Nuffield Department of Medicine, University of Oxford, Oxford, UK; 5 The Queen’s College, University of Oxford, Oxford, UK; 6 Department of Brain Repair & Rehabilitation, Queen Square Institute of Neurology, University College London, London, UK

**Keywords:** Dynamic susceptibility contrast (DSC) MR perfusion, Isocitrate dehydrogenase (IDH), 1p19q codeletion, Glioma, Neuro-oncology, Systematic review, Meta-analysis

## Abstract

**Purpose:**

Isocitrate dehydrogenase (IDH) mutation and 1p19q codeletion status are important for managing glioma patients. However, current practice dictates invasive tissue sampling for histomolecular classification. We investigated the current value of dynamic susceptibility contrast (DSC) MR perfusion imaging as a tool for the non-invasive identification of these biomarkers.

**Methods:**

A systematic search of PubMed, Medline, and Embase up to 2023 was performed, and meta-analyses were conducted. We removed studies employing machine learning models or using multiparametric imaging. We used random-effects standardized mean difference (SMD) and bivariate sensitivity-specificity meta-analyses, calculated the area under the hierarchical summary receiver operating characteristic curve (AUC) and performed meta-regressions using technical acquisition parameters (e.g., time to echo [TE], repetition time [TR]) as moderators to explore sources of heterogeneity. For all estimates, 95% confidence intervals (CIs) are provided.

**Results:**

Sixteen eligible manuscripts comprising 1819 patients were included in the quantitative analyses. IDH mutant (IDHm) gliomas had lower rCBV values compared to their wild-type (IDHwt) counterparts. The highest SMD was observed for rCBV_mean_, rCBV_max_, and rCBV 75^th^ percentile (SMD≈ − 0.8, 95% CI ≈ [− 1.2, − 0.5]). In meta-regression, shorter TEs, shorter TRs, and smaller slice thicknesses were linked to higher absolute SMDs. When discriminating IDHm from IDHwt, the highest pooled specificity was observed for rCBV_mean_ (82% [72, 89]), and the highest pooled sensitivity (i.e., 92% [86, 93]) and AUC (i.e., 0.91) for rCBV 10^th^ percentile. In the bivariate meta-regression, shorter TEs and smaller slice gaps were linked to higher pooled sensitivities. In IDHm, 1p19q codeletion was associated with higher rCBVmean (SMD = 0.9 [0.2, 1.5]) and rCBV 90^th^ percentile (SMD = 0.9 [0.1, 1.7]) values.

**Conclusions:**

Identification of vascular signatures predictive of IDH and 1p19q status is a novel promising application of DSC perfusion. Standardization of acquisition protocols and post-processing of DSC perfusion maps are warranted before widespread use in clinical practice.

**Supplementary information:**

The online version contains supplementary material available at 10.1007/s00234-023-03154-5.

## Introduction

Gliomas represent the most common primary brain tumors [1]. Constant classification updates from the World Health Organization (WHO) reflect progressive insights into the biological behavior of this heterogeneous group of tumors [2]. Thus, the early histological division into low-grade (LGGs) and high-grade gliomas (HGGs) was gradually replaced by categories based on histological features and molecular biomarkers such as isocitrate dehydrogenase (IDH) mutation and 1p19q codeletion status [3]. In view of the importance of the glial genotype for patient management and prognosis, the current classification (WHO 2021) attributes a pivotal role to IDH and 1p19q status for the discrimination between tumor subtypes (astrocytoma, IDH mutant [IDHm]; oligodendroglioma, IDHm and 1p19q codeleted; and glioblastoma, IDHm or IDH wild-type [IDHwt]) [2]. Thus, the identification of these biomarkers becomes crucial in the current landscape of clinical management of glioma patients.

The gold standard for establishing the IDH and 1p19q status is immunohistochemistry and genomic sequencing of histopathological tissue samples [4]. However, brain biopsy is invasive and despite recent advances remains prone to potential complications and sampling errors due to tumor heterogeneity [5]. Thus, an alternative non-invasive tool to accurately predict IDH and 1p19q status within a whole tumor sample would be of high clinical value. To this end, different MRI modalities have been explored to non-invasively characterize IDH mutation and 1p19q codeletion status in gliomas [6]. These include conventional MRI, diffusion-weighted imaging (DWI), magnetic resonance spectroscopy (MRS), and MR perfusion including dynamic susceptibility contrast (DSC). The advent of machine learning for multiparametric MRI analysis has further expanded the capabilities of imaging for molecular subtyping [7–9].

Recent meta-analyses demonstrate a good performance for conventional MRI [6], MRS [10], amide proton transfer [11], dynamic contrast enhanced (DCE)/DSC MR perfusion [12], and MRI radiomics [13] for molecular classification. Although many individual studies employed DSC MR perfusion for the molecular characterization of gliomas, no systematic review or meta-analysis focused specifically on the potential methodological limitations of the technique. With the advent of novel treatments including IDH inhibitors [14, 15], the value of DSC MR perfusion may extend further than pre-treatment genotyping of gliomas, allowing treatment selection and surveillance through the identification of genotype-driven vascular signatures. Furthermore, at a time in which machine learning is transforming this field via the integrated analysis of multiple modalities, scrutiny of the constitutional components of multiparametric MRI is imperative.

Our study aimed to investigate the added benefit of DSC MR perfusion for molecular subtyping of gliomas with an emphasis on methodological quality and moderators of classification performance. Therefore, studies applying multiparametric MRI analysis or machine learning were considered out of its scope. Specifically, this systematic review explored whether DSC perfusion can distinguish: (1) IDHm from IDHwt gliomas, (2) IDHm with 1p19q codeletion and IDHm without 1p19qcodeletion, and (3) investigate sources of heterogeneity and potential areas for methodological improvement. These were evaluated through random-effects standardized mean difference (SMD) and bivariate sensitivity–specificity meta-analyses. In addition, we performed meta-regressions to explore which DSC acquisition parameters and applied perfusion metrics associate with better classification performance.

## Methods

This systematic review was conducted in line with the Preferred Reporting Items for Systematic Reviews and Meta-analysis (PRISMA) criteria [16]. Each step was conducted independently by two reviewers (LS and CT) and any discrepancy was evaluated by a third senior reviewer (SB).

### Search strategy

The research question was “what is the current performance of DSC MR perfusion in identifying IDH mutation and 1p19q codeletion status in glioma patients?”. To answer this question, a systematic literature search was performed on PubMed, Medline, and Embase to find relevant publications between 01/01/2000 and 01/01/2023. The patient/intervention/comparator/outcomes (PICO) framework was used to define the search items: (P) = glioma, glioblastoma, brain tumor/tumor, brain neoplasia/neoplasm; (I) = dynamic susceptibility contrast, DSC, MR perfusion, brain perfusion; (C) = biopsy, histopathology, molecular profile, isocitrate dehydrogenase/IDH, 1p19q codeletion (and common variations such as 1p/19q, 1p-19q, 1p 19q, codeletion, codeleted); and (O) = predict, identify, classify, stratify, subtype, categorize, characterize, assign, cluster, distinguish. The PICO framework categories were combined using “AND,” while variations within categories were combined via “OR.” The same search items combinations were used for all databases. Reference lists were also reviewed to identify further eligible publications.

### Inclusion and exclusion criteria

The study selection process was conducted in EndNote X9. Eligibility criteria included peer-reviewed, English-written publications available through electronic indexation satisfying the following conditions: (1) DSC was used as an index test to determine IDH mutation and/or 1p19q codeletion status; (2) histopathology with molecular profiling was employed as the reference standard; (3) all patients received both the index test and reference standard within a reasonable timeframe with blinding of the evaluators.

Exclusion criteria were (1) studies employing multiparametric imaging, (2) the use of regression models incorporating additional covariates (e.g., sex, age) to predict IDH and 1p19q status, (3) studies using machine learning, (4) very small studies (< 10 glioma patients), (5) pediatric studies, and (6) non-original research (e.g., case-reports, reviews, abstracts, conference presentations, book chapters).

### Data extraction

LS and CT independently extracted data from both the manuscripts as well as the supplementary materials of the included studies using a standardized Microsoft Excel 2016 spreadsheet previously agreed by all authors. We extracted data on (1) study design (retrospective vs. prospective), (2) participant characteristics (i.e., mean age, sex ratio, and ethnical breakdown), (3) glioma characteristics (i.e., grade and stage), (4) DSC imaging acquisition parameters (e.g., time to echo [TE], repetition time [TR], pulse sequence, field of view, slice thickness, slice gap, use of contrast preload, contrast dose), and (5) prefusion map post-processing (e.g., region of interest [ROI] selection, leakage correction, standardization/normalization methodology). In addition, we extracted DSC metrics (e.g., relative cerebral blood volume [rCBV]) in IDHwt, IDHm, IDHm with 1p19q codeletion and IDHm without 1p19q codeletion groups. Finally, we recorded the diagnostic accuracy for the prediction of IDH and 1p19q status (e.g., sensitivity, specificity, positive predictive value, negative predictive value). In general, there was a good agreement between LS and CT for both categorical (Cohen’s Kappa 0.88) and continuous variables (intraclass correlation coefficient: 0.92). The input of a senior reviewer (SB) was sought to resolve discrepancies.

### Quality assessment

This systematic review’s quality was assured by the Quality Assessment of Diagnostic Accuracy Studies 2 (QUADAS-2) questionnaire in its original format [17]. Patient selection, study conduct, and interpretation of IDH and 1p19q status by DSC and histopathological analysis with molecular profiling were appraised in line with the review question exploring applicability concerns and risks of bias.

### Statistical methods

All statistical analyses were performed using R version 4.2.2 (packages “meta,” “metaphor,” and “mada”). A *p*-value < 0.05 was regarded as significant.

Heterogeneity between studies was assessed using Cochran’s *Q* and Higgins *I*^2^ statistics. A Cochran’s *Q* test with a *p*-value < 0.05 or *I*^2^ > 50% were interpreted as potentially indicative of the presence of heterogeneity [18]. Regardless of the degree of heterogeneity, a random-effects model was used for all meta-analyses. In addition, meta-regression was conducted to explore sources of heterogeneity in any meta-analysis including at least 4 studies. Evaluated moderator variables included continuous (contrast dose, TE, TR, slice thickness, slice gap, acquisition time) and categorical variables (contrast type, pulse sequence, ROI selection, DSC standardization/normalization, analysis software, arterial input function [AIF] and the use of leakage correction).

Publication bias was assessed via the visual inspection of contour-enhanced funnel and Egger’s test. Asymmetrical funnel plots or Egger’s test *p* < 0.05 were interpreted as potentially indicating the possibility of publication bias [19].

### Standardized mean difference meta-analysis

We performed comparisons between IDHm and IDHwt for different combinations of WHO grades including grade 2, grade 3, grade 4, and across all WHO grades. In addition, we compared IDHm with 1p19q codeletion vs. IDHm without 1p19q codeletion. Firstly, we extracted the mean, standard deviation (SD), and the number of glioma patients in each of the groups described above for each available DSC metric. As the measurement scales differed between the studies, we performed the comparisons by calculating the SMDs and their 95% confidence intervals [CIs] [20].

### Diagnostic accuracy meta-analysis

In this analysis, we included all studies which reported enough metrics enabling the computation of true positives (TP), true negatives (TN), false positives (FP), and false negatives (FN).

We used validated diagnostic test accuracy meta-analysis methodology to calculate the pooled sensitivity and specificity [21]. Given the inter-dependence of sensitivity and specificity, a bivariate model was used to generate the hierarchical summary receiver operating characteristic (HSROC) curve with its 95% confidence region and to compute the area under the ROC curve (AUC) [22].

In addition, we computed the pooled diagnostic odds ratios (DORs), positive likelihood ratios (PLRs), and negative LRs (NLRs) and their 95% CIs. DOR represents the odds of the IDH mutation being correctly identified by DSC. PLR captures the ratio of the probability that DSC predicts an IDH mutation in IDHm vs IDHwt (i.e., LR for positive results). In contrast, NLR represents the LR for negative results.

## Results

### Database search

**Fig. 1:**
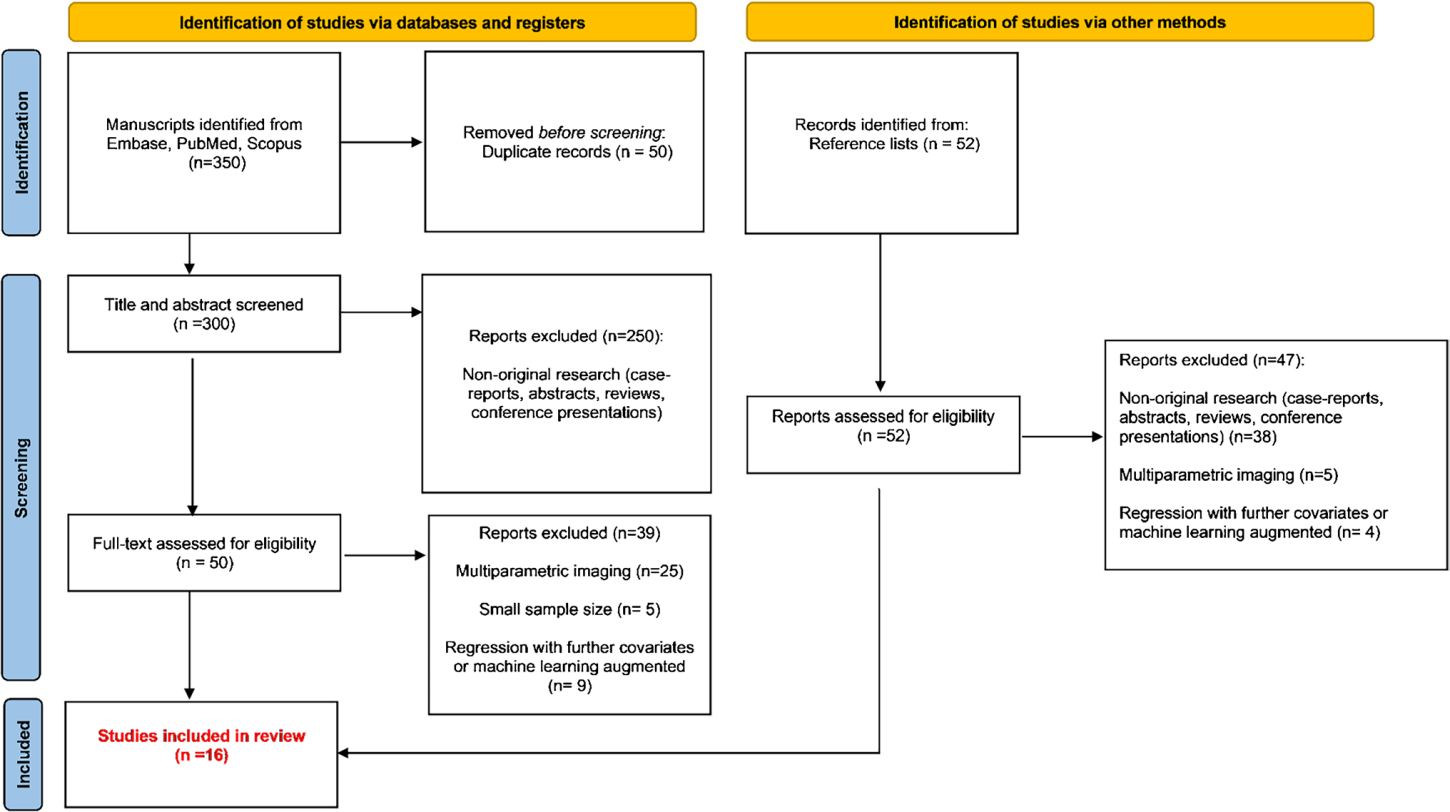
Preferred Reporting Items for Systematic Reviews and Meta-analysis (PRISMA) flow chart of study selection process

A PRISMA flow chart of the database search process is presented in Fig. 1. Briefly, the initial search identified 352 potential articles. After removing duplicates and screening abstracts against our research question, we further considered 102 papers. Following the application of our inclusion and exclusion criteria, 16 manuscripts were included in the quantitative analysis [23–38]. The characteristics of these studies were extracted and tabulated in Table 1.

**Table 1:** Study characteristics

Study*	IDH mutant: wild-type ratio	1p19q codeletion	WHO grade2: grade3: grade4	DSC TE/TR (ms)	DSC pulse sequence	DSC field of view (mm x mm)	DSC slice thickness/gap (mm/mm)	DSC normalization	Arterial input function	Leakage correction (including pre-bolus)	Contrast dose (mmol/kg/rate (ml/s)	ROI selection
Brendle 2020 [23]	32:24	16	29:30:7	31/1130	Gradient-echo echo-planar	230 × 230	4/N/A	Normalized	Manual	Yes	0.1/3	Manual: tumor
Choi 2019 [24]	125:338	49	32:142:289	29.3–40/1500–1600	T1-weighted 3D magnetization-prepared rapid acquisition gradient-echo (MPRAGE)	220 × 220 to 240 × 240	5–6/0.9–1.5	Standardized	Automatic	No	N/A	Automatic: tumor (cMRI co-registration)
Cindil 2022 [25]	23:35	N/A	0:23:35	30/1500	N/A	230 × 230	5/1.5	Normalized	N/A	No	0.1/5	Manual: hotspot
Guo 2022 [26]	54:48	N/A	37:22:43	40/1700	N/A	N/A	6/0	Normalized	N/A	N/A	0.2/3.5	Manual: hotspot
Hempel 2019 [27]	54:46	23	40:30:30	31/1130	Gradient-echo echo-planar	230 × 330	4/N/A	Normalized	Manual	Yes	0.1 (× 2)/3.5	Manual: tumor
Kickingereder 2015 [28]	60:13	N/A	34:49:0	36/2220	Gradient-echo echo-planar	240 × 240	5/N/A	Standardized	Automatic	Yes	0.1/ N/A	Automatic: tumor (cMRI co-registration)
Lee 2015 [29]	16:36	N/A	0:36:16	30–40/1500	Gradient-echo echo-planar	240 × 240	5/1	Normalized	N/A	Yes	0.1/4	Manual: tumor
Lee 2020 [30]	50:45	46	45:65:0	40/1808	Gradient-echo echo-planar	240 × 240	5/2	Normalized	N/A	Yes	0.1/4	Manual: hotspot
Lee_MH 2019 [31]	12:76	NA	0:0:88	35/1720	Gradient-recalled T2*-weighted echo-planar		5/N/A	N/A	N/A	Yes	N/A	Manual: hotspot
Song 2021 [32]	22:30	13	16:6:30	15.4/1800	Gradient-recalled T2*-weighted echo-planar	240 × 240	5/1	Normalized	Automatic	Yes	0.1/4	Manual: tumor
Tan 2017 [33]	32:59	N/A	31:24:36	30/1500	Gradient-echo echo-planar	230 × 230	4/1.2	Normalized	Manual	N/A	N/A / 3.5	Manual: hotspot
Wu 2020 [34]	19:25	10	0:19:25	30/1500	Gradient-echo echo-planar	240 × 240	5/1	Normalized	N/A	Yes	0.1/4	Manual: tumor
Xing 2017 [35]	17;25	N/A	24:18:0	54/1000–1250	Gradient-recalled T2*-weighted echo-planar	220 × 220	5/1	Normalized	Automated	No	0.1/5	Manual: hotspot
Xing 2019 [36]	10:65	N/A	0:0:75	54/1000–1250	Gradient-recalled T2*-weighted echo-planar	220 × 220	5/1	Normalized	Automated	No	0.1/5	Manual: tumor
Yang 2021 [37]	142:0	73	N/A	54/1000–1250	Gradient-recalled T2*-weighted echo-planar	220 × 220	5/1	Normalized	Automated	No	0.1/5	Manual: tumor
Zhang 2020 [38]	23:20	N/A	14:14:15	30/1600	Gradient-echo echo-planar	220 × 220	4/N/A	Normalized	Manual	N/A	0.1/3.5	Manual: tumor

All studies except Wu 2020 were retrospective

All studies except Choi 2019 used a field strength of 3 T. Choi 2019 used both 1.5 T and 3 T scanners

All studies except Choi 2019 (100 × 100 to 128 × 128), Lee MH 2019 (80 × 96 to 128 × 128), Song 2021 (96 × 128) used a DSC matrix of 128 × 128

All regions of interest (ROIs) or volume of interests (VOIs) avoided cystic, necrotic, haemorrhagic, and areas containing macrovessels. “Tumor” refers to the entire enhancing region identified on post contrast sequences

*3D* three-dimensional, *cMRI* conventional magnetic resonance imaging, *DSC* dynamic susceptibility contrast, *IDH* isocitrate dehydrogenase, *N/A* not available, *ROI* region of interest, *TE*, time to echo, *TR* repetition time, *WHO* World Health Organization

### Quality assessment

Overall, the 16 studies included in the quantitative analysis were of a high methodological quality.

**Fig. 2:**
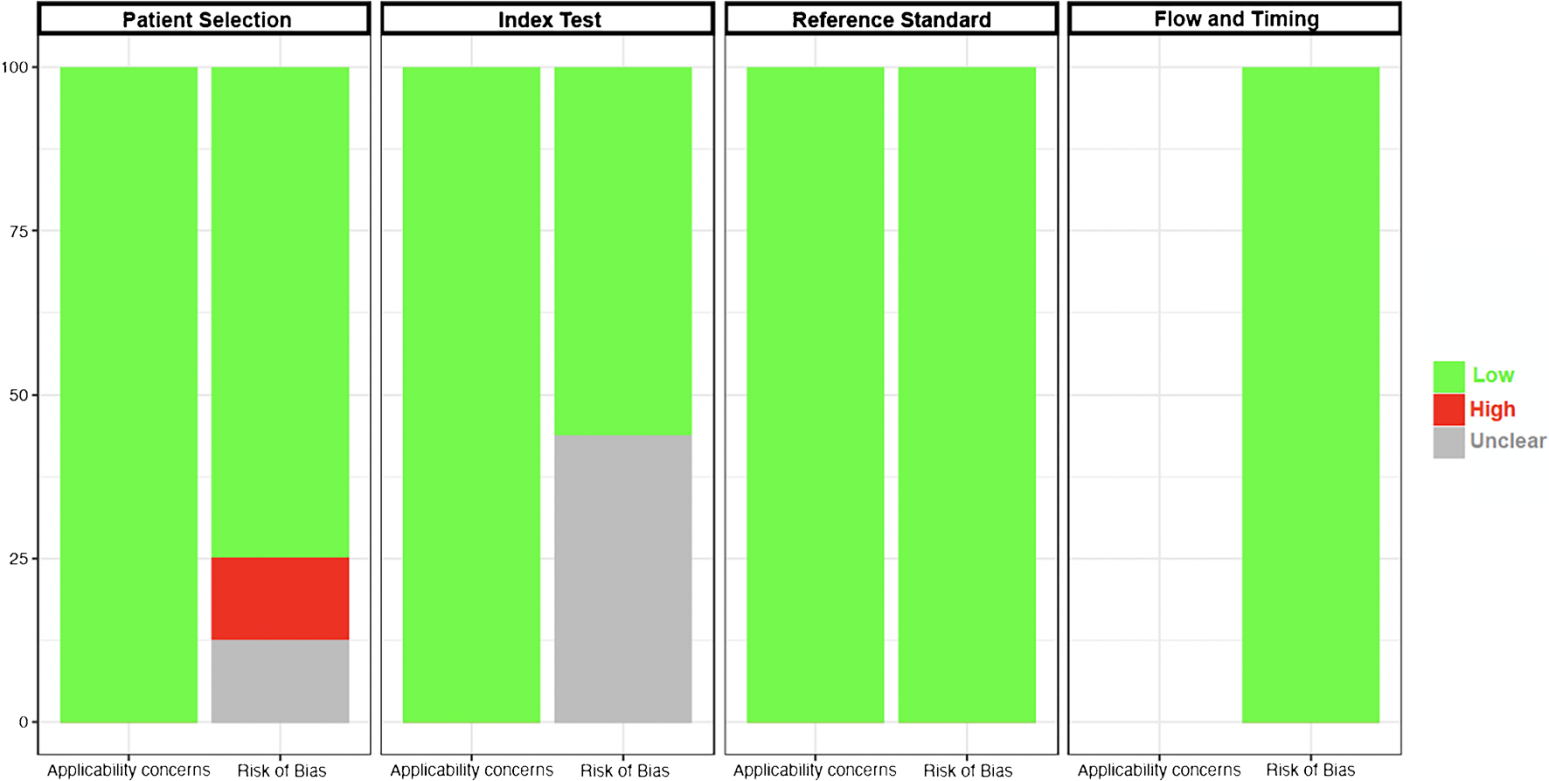
QUADAS-2 questionnaire: quality assessment results. According to QUADAS-2, risk of bias is assessed across four domains (patient selection, index test, reference standard, and flow and timing), while applicability concerns are assessed in only the first three domains. QUADAS-2 Quality Assessment of Diagnostic Accuracy Studies-2

The QUADAS-2 spreadsheet summarizing our evaluation for each questionnaire item for each of the included studies is provided in Supplementary Material 1. The risk of bias was evaluated in the patient selection, index test, reference standard, and flow and timing domains according to the QUADAS-2 questionnaire (Fig. 2). In the patient selection domain, 13% of studies did not provide enough information on their exclusion criteria and 13% might have made inappropriate exclusions (e.g., excluding individuals for whom a full molecular profile besides IDH status was not available). The risk of bias in the index test domain was assessed as unclear in 44% of studies, which did not explicitly state that neuroradiologists were blinded to IDH mutation status. Although 75% of studies did not specify the interval between the MR perfusion and biopsy, the risk of bias was considered low given that IDH mutation status is unlikely to change in a relatively short timeframe. Applicability concerns were also evaluated in the patient selection, index test, and reference standard domains. The were no applicability concerns by design as such studies would be filtered by our exclusion criteria.

### Differentiating IDHm from IDHwt regardless of WHO grade

The most commonly used DSC metric when comparing IDHm vs. IDHwt was rCBV_mean_. The SMD meta-analysis results are presented in Table 2, while the diagnostic accuracy meta-analysis results are summarized in Table 3.

**Table 2:** Standardized mean difference (SMD) meta-analysis

DSC metric	Number of studies	Sample size		Heterogeneity		Effect size		Egger’s test*
				*I*^2^	Cochran’s *Q* test *p*-value	SMD (95% CI)	*p*-value	*p*-value
		IDH mutant: all WHO grades	IDH wild-type: all WHO grades					
rCBV_mean_	8	379	635	77.95%	0.0002	− 0.80 (− 1.13, − 0.47)	** < 0.0001**	0.877
rCBV_median_	4	171	237	40.91%	0.150	− 0.52 (− 0.81, − 0.23)	** < 0.0001**	0.367
rCBV_max_	3	61	125	0.00%	0.416	− 0.80 (− 1.15, − 0.45)	** < 0.0001**	0.750
rCBV 95th	2	190	383	11.60%	0.288	− 0.64 (− 0.84, − 0.43)	** < 0.0001**	N/A
rCBV 90th	2	136	126	52.54%	0.120	− 0.73 (− 1.12, − 0.35)	** < 0.0001**	N/A
rCBV 75th	2	67	121	0.00%	0.324	− 0.81 (− 1.16, − 0.47)	** < 0.0001**	N/A
rCBV 25th	2	67	121	30.51%	0.230	− 0.71 (− 1.13, − 0.28)	**0.001**	N/A
rCBV 10th	2	71	81	83.77%	**0.013**	− 0.34 (− 1.23, 0.55)	0.457	N/A
		IDH mutant: WHO 2	IDH wild-type: WHO 2					
rCBV_mean_	3	66	37	72.23%	**0.027**	− 1.10 (− 1.98, − 0.22)	**0.015**	**0.017**
		IDH mutant: WHO 3	IDH wild-type: WHO 3					
rCBV_mean_	4	114	97	18.10%	0.174	− 0.52 (− 0.79, − 0.24)	**0.0003**	0.631
rCBV_median_	3	23	22	54.64%	0.111	− 0.10 (− 0.84, 0.64)	0.786	**0.046**
rCBV 90th	2	36	32	28.65%	0.237	− 0.57 (− 1.31, 0.17)	0.237	N/A
rCBV 10th	2	23	22	74.07%	**0.049**	− 0.02 (− 1.28, 1.24)	0.976	N/A
		IDH mutant: WHO 4	IDH wild-type: WHO4					
rCBV_mean_	4	47	337	19.08%	0.203	− 0.51 (− 0.89, − 0.13)	**0.009**	0.734
rCBV_median_	3	13	46	0.00%	0.536	− 0.36 (− 0.84, 0.12)	0.137	0.760
rCBV 90th	2	23	71	0.00%	0.850	− 0.60 (− 1.23, 0.03)	0.061	N/A
rCBV 10th	2	13	46	22.44%	0.256	− 0.39 (− 1.10, 0.33)		N/A
		IDH mutant: 1p 19q codeletion	IDH mutant: 1p 19q no codeletion					
rCBV_mean_	3	85	66	69.35%	**0.031**	0.89 (0.24, 1.53)	**0.007**	0.633
rCBV_median_	2	89	85	82.20%	**0.018**	0.77 (− 0.18, 1.71)	0.112	N/A
rCBV_max_	2	69	50	53.89%	0/141	0.30 (− 0.25, 0.84)	0.282	N/A
rCBV 90th	2	69	50	73.90%	0.050	0.92 (0.13, 1.71)	**0.022**	N/A

^*^Egger’s test was used only when there were more than two studies in that category

*CI* confidence interval, *rCBV* relative cerebral blood volume, *SMD* standardized mean difference. Other abbreviations as in Table 1

**Table 3:** Diagnostic accuracy meta-analysis

Marker	Studies	Sample size		Diagnostic accuracy									
				Sensitivity (95% CI)	*I*^2^	Cochran’s *Q* test *p*-value	Specificity (95% CI)	*I*^2^	Cochran’s *Q* test *p*-value	DOR (95% CI)	PLR (95% CI)	NLR (95% CI)	AUC
		IDH mutant: All WHO grades	IDH wild-type: All WHO grades										
rCBVmean	6	295	509	79.16 (57.30, 91.49)	90.3%	< 0.0001	82.32 (72.19, 89.31)	73.9%	< 0.0001	13.58 (8.10, 22.75)	3.91 (2.78, 5.51)	0.36 (0.24.0.54)	0.870
rCBV_median_	3	138	93	88.65 (72.63, 95.38)	68.7%	0.017	61. 59 (37.65, 80.99)	75.4%	0.002	12.04 (1.66, 87.65)	2.27 (1.08, 4.76)	0.21 (0.06, 0.74)	0.839
rCBV 90th	3	131	94	87.73 (62.13, 96.89)	82.6%	0.0006	68.75 (43.75, 86.15)	75.6%	0.005	12.79 (5.94, 27.56)	2.26 (1.50, 3.41)	0.21 (0.07, 0.61)	0.832
rCBV 75th	2	115	58	91.03 (80.28, 96.19)	42.1%	0.068	49.55 (36.99, 62.17)	0.0%	0.318	11.44 (1.68, 77.79)	1.78 (1.19, 2.65)	0.17 (0.04, 0.73)	0.745
rCBV 25th	2	115	58	79.47 (60.14, 90.85)	73.5%	0.007	74.35 (61.66, 83.94)	0.0%	0.552	11.13 (2.20, 56.24)	2.75 (1.73, 4.37)	0.27 (0.09, 0.82)	0.804
rCBV 10th	2	115	58	92.12 (85.58, 95.84)	0.0%	0.423	61.78 (32.97, 84.15)	57.8%	0.032	20.98 (2.34, 187.95)	2.56 (0.86, 7.60)	0.13 (0.04, 0.38)	0.911
		IDH mutant: WHO 2	IDH wild-type: WHO 2										
-	-	-	-	-	-	-	-	-	-	-	-	-	-
		IDH mutant: WHO 3	IDH wild-type: WHO 3										
rCBV_mean_	2	19	34	83.17 (59.77, 94.27)	0.0%	0.698	95.32 (74.31, 99.31)	9.0%	0.999	50.09 (8.33, 301.32)	8.51 (2.38, 30.39)	0.22 (0.09, 0.51)	0.876
		IDH mutant: WHO 4	IDH wild-type: WHO4										
rCBV_mean_	2	15	55	82.07 (55.03, 94.48)	0.0%	0.919	96.36 (69.08, 99.68)	45.8%	0.999	47.77 (6.64, 343.57)	11.47 (1.81, 72.86)	0.24 (0.10, 0.59)	0.84

*-* analysis not possible, *AUC* area under the hierarchical summary receiver operating characteristic curve, *DOR* diagnostic odds ratio, *NLR* negative likelihood ratio, *PLR* positive likelihood ratio. Other abbreviations as in Table 2

**Fig. 3:**
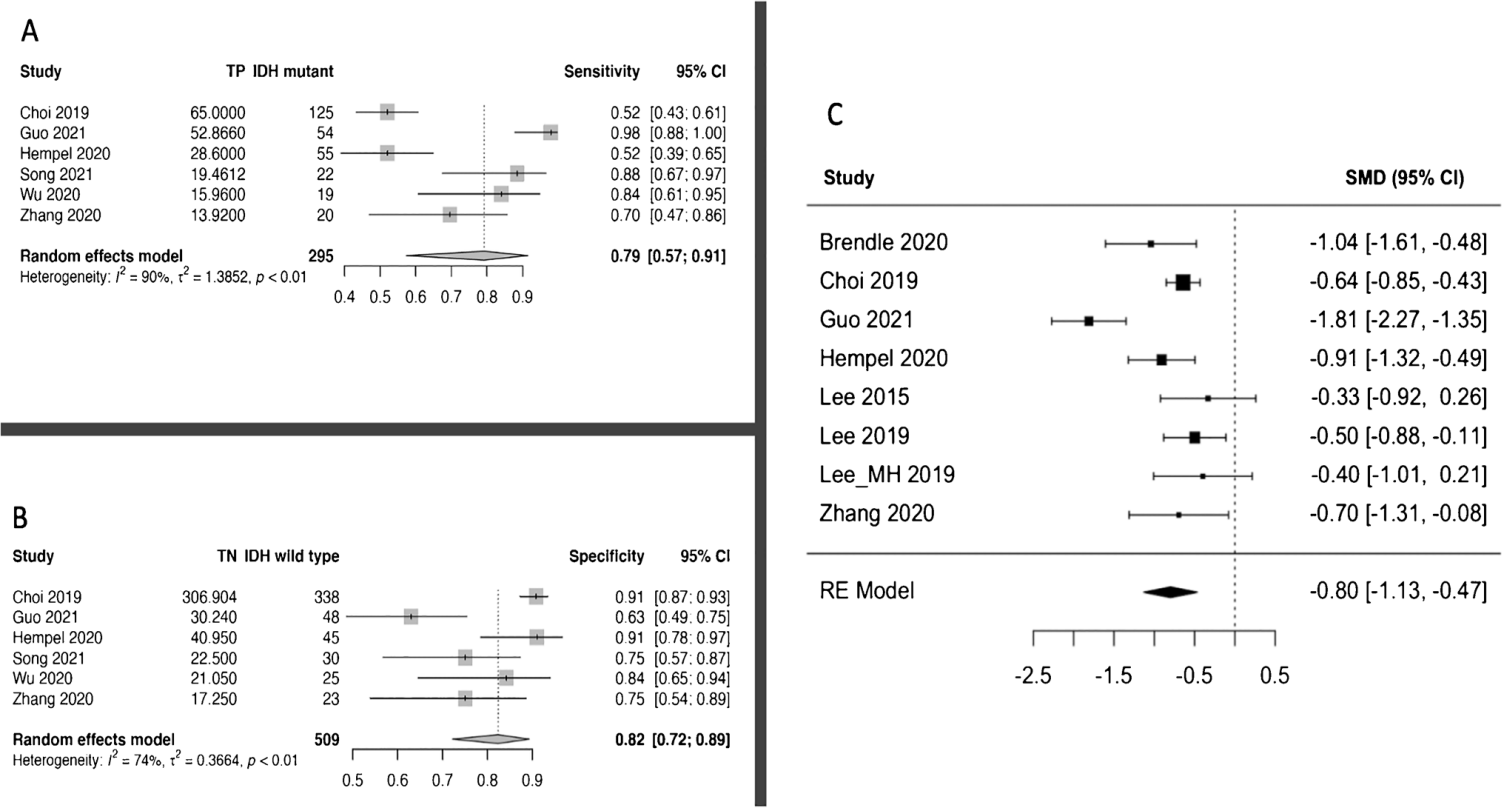
Forest plots for the random-effects rCBVmean meta-analyses of IDH mutant vs. IDH wild-type gliomas for: **A** sensitivity, **B** specificity, and **C** SMD (panel **C**). CI, confidence interval; IDH, isocitrate dehydrogenase; rCBV, relative cerebral blood volume; *p*, *p*-value; RE, random-effects; SMD, standardized mean difference; TN, true negative; TP, true positive

When attempting to differentiate IDHm from IDHwt, the highest pooled sensitivity was achieved by rCBV 10^th^ percentile (92%, 95% CI: [86, 93]) (Table 3), and the highest pooled specificity by rCBVmean (82% [72, 89]) (Fig. 3). Although the highest diagnostic performance was observed for rCBV 10^th^ percentile (DOR = 20.96 [2.34–187.95], AUC = 0.91), this analysis included only 2 studies. In general, the heterogeneity between the studies was substantial (i.e., *I*^2^ > 50%, Cochran’s *Q p* < 0.05) except for rCBV 75^th^ percentile sensitivity (*I*^2^ = 42%, *p* = 0.068) and specificity (*I*^2^ = 50%, *p* = 0.318), rCBV 25^th^ percentile specificity (*I*^2^ = 0%, *p* = 0.552) and rCBV 10^th^ percentile sensitivity (*I*^2^ = 0%, *p* = 0.423) analyses.

**Fig. 4:**
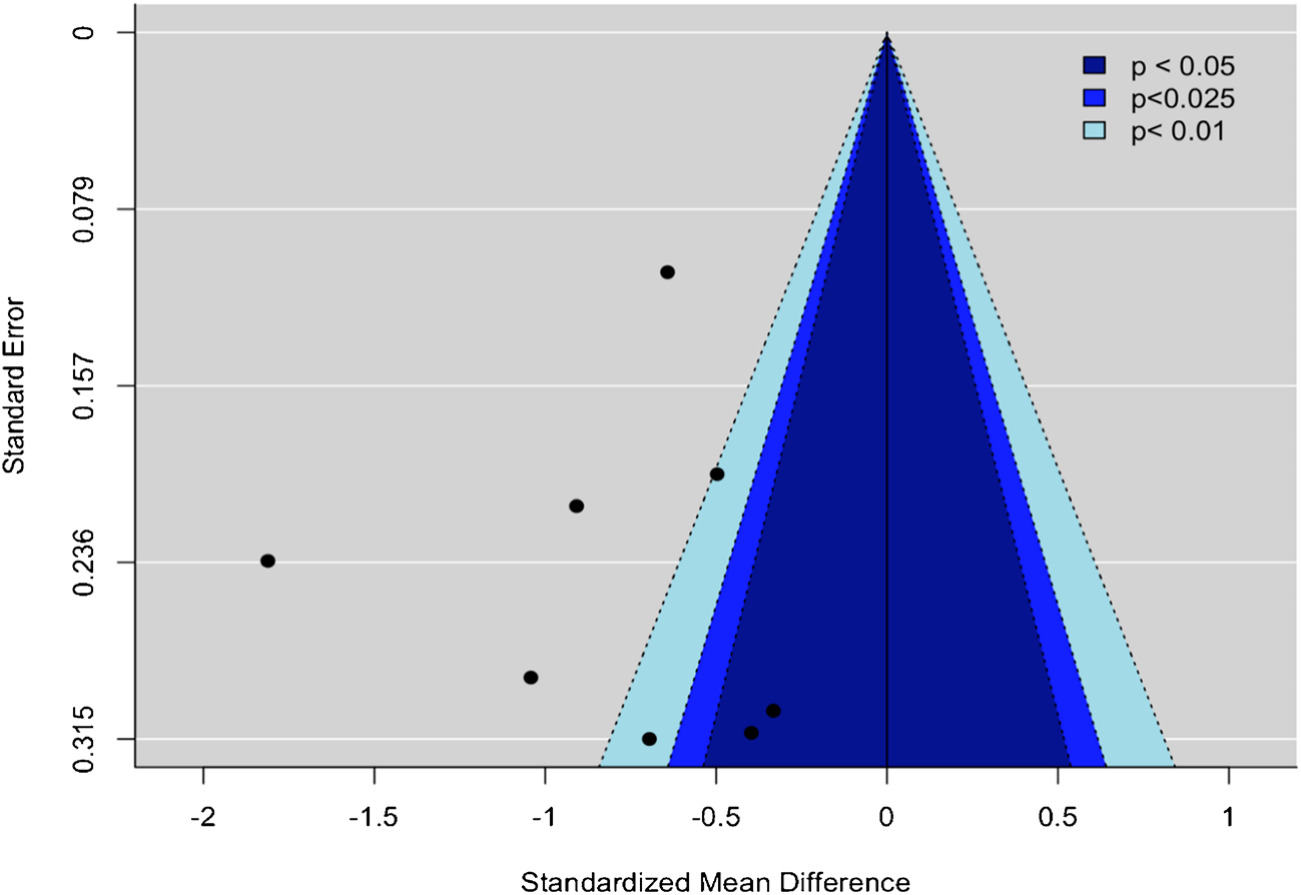
Contour-enhanced funnel plot for the standardized mean difference rCBV_mean_ meta-analysis of IDH mutant gliomas vs. IDH wild-type gliomas. Abbreviations as in Fig. 3

**Fig. 5:**
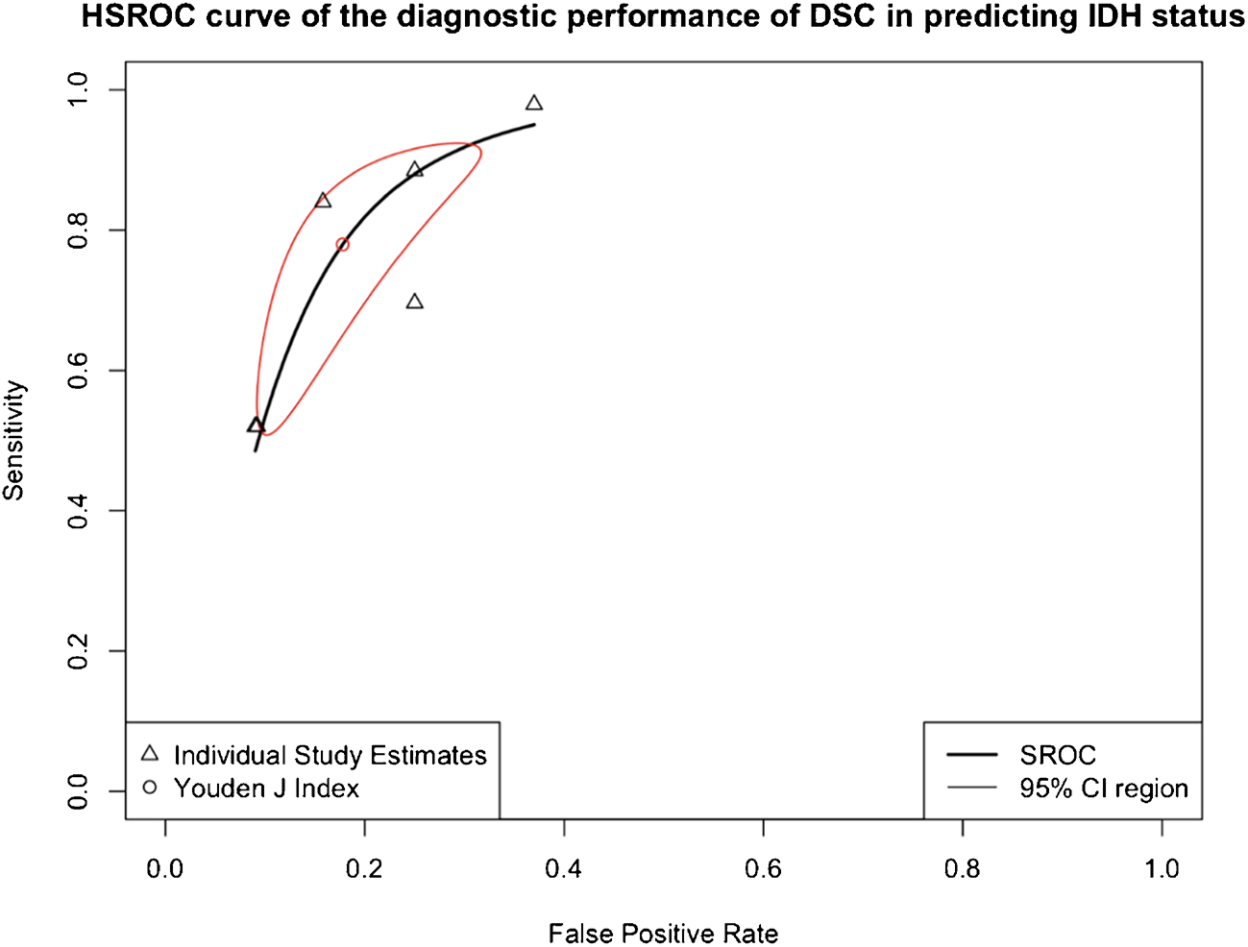
HSROC curve of the diagnostic performance of rCBVmean in differentiating IDH mutant gliomas vs. IDH wild-type gliomas. False-positive rate is plotted against the sensitivity for all studies to yield the hierarchical summary receiver operating characteristic curve. Youden’s *J* index represents the optimum cut-off between sensitivity and specificity. The 95% confidence region of the diagnostic performance of rCBV_mean_ in differentiating IDH mutant from IDH wild-type gliomas is presented in red*.* HSROC, hierarchical summary receiver operating characteristic. Other abbreviations as in Fig. 3

According to the SMD meta-analysis, IDHm consistently had higher rCBV values across all DSC metrics compared to their IDHwt counterparts except for rCBV 10^th^ percentile where the difference was not statistically significant. The highest SMDs were observed for rCBV_mean_ (SMD =  − 0.8 [− 1.1, − 0.5]) (Fig. 3), rCBV_max_ (SMD =  − 0.8 [− 1.2, − 0.5]), and rCBV 75^th^ percentile (SMD =  − 0.8 [− 1.2, − 0.5]) (Table 2). In contrast to the sensitivity and specificity meta-analyses, heterogeneity was generally low except for the rCBV_mean_ (*I*^2^ = 78%, *p* < 0.001) and rCBV 10^th^ percentile (*I*^2^ = 84%,* p* = 0.013) analyses. In general, the Egger tests and the visual inspection of the contour-enhanced funnel plots suggested a low possibility of publication bias (Figs. 4 and 5).

### Differentiating IDHm from IDHwt in WHO grade subgroups

The SMD meta-analysis results are presented in Table 2, and the diagnostic accuracy meta-analysis results are summarized in Table 3.rCBV_mean_ was lower in grade 2 IDHm compared to grade 2 IDHwt tumors (SMD =  − 1.1 [− 2.0, − 0.2]), however with considerable heterogeneity (*I*^2^ = 72%, *p* = 0.027) and possibility of publication bias (Egger test *p*-value = 0.017).

In grade 3 gliomas, rCBV_mean_ was lower in IDHm (SMD =  − 0.5 [− 0.2, − 0.8]). For predicting IDH status, rCBV_mean_ yielded a pooled sensitivity of 83% [60, 94], a pooled specificity of 95% [74, 99], and an AUC of 0.88. The heterogeneity and possibility of publication bias were low.

In grade 4 gliomas, rCBV_mean_ differentiated IDHm from IDHwt with a pooled sensitivity of 82% [55, 95] and pooled specificity of 96% [69, 100] yielding an AUC = 0.84. Indeed, rCBV_mean_ was lower in IDHm vs IDHwt (SMD =  − 0.5 [− 0.9, − 0.1]). The heterogeneity and possibility of publication bias were also low.

### Differentiating IDHm with 1p19q codeletion from IDHm without 1p19q codeletion

The SMD meta-analysis results are presented in Table 2. A diagnostic accuracy meta-analysis was not performed due to a lack of studies. IDHm with 1p19q codeletion demonstrated higher rCBV_mean_ compared to IDHm without 1p19q codeletion (SMD = 0.9 [0.2, 1.5]). A higher rCBV 90^th^ percentile was also observed in IDHm with 1p19q codeletion (SMD = 0.9 [0.1, 1.7]). Heterogeneity between the studies was high (all *I*^2^ > 50%, all *p* < 0.05) albeit the possibility of publication bias was low (Egger test *p*-value = 0.633).

### Meta-regression

The meta-regression results for the SMD analysis are presented in Supplementary Table S1, and for the diagnostic accuracy analysis in Supplementary Table S2 from Supplementary Material 2.

Shorter TEs were associated with higher absolute SMDs for rCBV_mean_ (i.e., greater absolute difference between IDHm and IDHwt) which translated into higher pooled sensitivities (for a 1 ms decrease in TE, the sensitivity increased by 0.1) in the bivariate sensitivity-specificity model. Similarly, shorter TRs increased the absolute SMDs between IDHm and IDHwt for rCBV_mean_ (for a 1 ms increase in TR, the absolute value of SMD increased by 0.001), however without significant increase in diagnostic accuracy (both *p* > 0.100). Smaller slice thicknesses were associated with higher absolute SMDs for rCBV_median_, while smaller slice gaps were associated with higher pooled sensitivities and specificities for rCBV_mean_.

In general, DSC metrics were usually calculated either based on manual hotspot or manual total enhancing tumor area ROIs. Studies using hotspot ROIs obtained a higher sensitivity (98% [87, 100]) at the cost of lower specificity (63% [49, 75]) for distinguishing IDHm from IDHwt using rCBV_mean_.

No other moderator was associated with study heterogeneity in any of the remaining meta-regressions.

## Discussion

This study suggests that DSC MR perfusion has a promising diagnostic performance for the non-invasive prediction of IDH mutation and 1p19q codeletion status in gliomas. Despite the inter-study heterogeneity, most studies reported sensitivities > 85% and specificities > 75% in distinguishing IDHm from IDHwt. For IDH mutation prediction, the most widely employed metric namely normalized rCBV_mean_, yielded a pooled sensitivity of 79% [57–92] and a polled specificity of 82% [72–89] respectively. IDHm had a lower rCBV_mean_ compared to their wild-type counterparts across all WHO grades. In IDHm, 1p19q codeletion associated with a higher rCBV_mean_ compared to 1p19q retained tumors. Estimation of cut-off values for DSC metrics was not possible based on our analysis given the heterogeneity in methodology. As a step towards protocol standardization, we describe acquisition parameter trends associated with an increased discriminatory performance: shorter TEs, shorter TRs, smaller slice thickness, and smaller slice gaps.

MR perfusion is well suited to predict IDH and 1p19q status in gliomas as it captures their genetically determined vascular habitats. Indeed, Kickingereder et al. [28] identified a link between specific hypoxia-angiogenesis transcriptome signatures and vascular phenotypes on DSC perfusion. IDHm were shown to have decreased activation of the hypoxia inducible factor 1A, which in turn rendered these tumors less prone to hypoxia induced angiogenesis compared to their wild-type counterparts. Therefore, decreased rCBV_mean_ values are expected in IDHm versus IDHwt, as demonstrated by multiple studies and confirmed by our meta-analysis.

The propensity of DSC perfusion to identify such vascular phenotypes may allow clinical applications extending beyond pre-treatment stratification, to treatment selection and post-treatment surveillance. Despite current trends favoring surgical management of gliomas, the advent of novel targeted treatments including IDH inhibitors, holds promise for a future paradigm shift. Brain-penetrant IDH inhibitors such as vorasidenib showed tumor suppressing effects in laboratory studies and demonstrated adequate safety in phase 1 clinical trials [14, 15, 39]. In addition, a vaccine with an IDH1 epitope has been developed [40] and demonstrated safety in clinical trials, making it an exciting potential therapy for IDHm tumors [41]. In this context, non-invasive prediction of IDH mutation status could aid treatment selection, especially for inoperable tumors such as brainstem gliomas [42]. Similarly, prediction of 1p19q codeletion status would favor patient stratification and prognostication, according to the current clinical management guidelines [43]. Genotype-driven perfusion signatures can be monitored during treatment, enabling assessment of new therapies in clinical trials and post-treatment, improving surveillance of patients. Recent studies report IDH dependent vascular trends revealing tumor recurrence using DSC perfusion [44, 45], which could partly explain shortcomings in predicting disease progression based on prior classifications.

## Comparative diagnostic performance

Multiple studies support the value of MRI for molecular classification of gliomas. DSC perfusion performs within the range of most investigated techniques, however, lacks in diagnostic performance compared to MRS. According to a systematic review [6], brain MRI yields a pooled sensitivity and specificity of 86% [79, 91] and 87% [78, 92] respectively. However, the review included studies employing different MRI techniques (e.g., MR diffusion, MR perfusion) with heterogeneous acquisition and post-processing protocols. A recent meta-analysis suggested that both DCE and DSC perfusion have a similarly satisfactory performance in establishing IDH mutation status with the highest AUC obtained for CBV (0.85 [0.75–0.93]) and extravascular space (Ve; 0.84 [0.71–0.97]) [46]. Our findings on the performance of DSC perfusion are consistent with the results published in this meta-analysis. However, our analysis addressed areas not reported by the latter study including (1) pooled sensitives and specificities for glioma classification, (2) pooled estimates for each DSC perfusion metric, and (3) meta-regression of performance moderators. Despite the promising results on the use of MR perfusion, the highest diagnostic performance to date was reported for 2-hydroxyglutarate (2HG) MRS which was shown to have a pooled sensitivity of 95% [85, 98] and specificity of 91% [83, 96] across 14 studies [10].

Despite differences in performance compared to MRS, we propose that DSC perfusion holds promise for predicting IDH and 1p19q status in gliomas, and that the value of these modalities lies not in their differences but in their complementary nature. Specifically, while MRS allows direct characterization of IDH mutation status via the identification of downstream biomarkers (e.g., 2HG), DSC perfusion identifies vascular phenotypes associated with this mutation. Both features have been independently proven to hold clinical value for pre-treatment stratification and post-treatment surveillance. The potential value of combining these techniques has been highlighted by previous studies on glioma grading [47, 48]. Therefore, the complementary nature of these modalities may offer unprecedented capabilities for pre-treatment patient stratification and especially for post-treatment surveillance. To this end, the combined techniques may allow longitudinal assessment of tumor cellularity and oncometabolites by MRS, and changes in tumor vascularity by DSC perfusion. Furthermore, although 2HG MRS demonstrates a good performance, the technique also poses challenges related to acquisition and post-processing [49]. Such limitations potentially surpass those of DSC perfusion, with recent studies reporting wider clinical adoption of DSC compared to MRS [50]. Our meta-regression analysis also indicates potential underestimation of DSC perfusion performance attributed to technical heterogeneity and choice of perfusion metrics, and highlights areas for improvement.

### Heterogeneity–standardization

Our study highlights the lack of DSC MR perfusion protocol standardization and suggests specific technical parameters contributing to heterogeneity. Specifically, shorter TEs and smaller slice gaps were associated with higher sensitivities in the bivariate sensitivity-specificity model, while shorter TEs, shorter TRs, and smaller slice thicknesses were linked to higher absolute SMDs between IDHm and IDHwt. The need for standardization of DSC MR perfusion is widely recognized, and our results highlight important technical considerations for improved diagnostic performance. Across both the SMD and diagnostic performance meta-regressions, TE was the only consistent metric associated with heterogeneity. The effects of TE in the quality of quantitative analysis of DSC perfusion have been previously described. In accordance with our findings, previous studies highlighted the limitations of long TEs likely due to saturation of the arterial input signal. To overcome this limitation, dual-echo and more recently multi-echo techniques have been proposed for the optimization of DSC perfusion [51, 52]. However, TE influences DSC perfusion in conjunction with multiple parameters, including flip angle, slice thickness, and contrast pre-load [52–54]. Using DSC perfusion simulations, Leu et al. [54] examined the effects of such acquisition parameters to the “faithful” estimation of rCBV. In an exploratory manner, we assessed the rCBV estimation error of the included papers with adequate protocol information, based on the published heat map diagram [54]. Based on this predictive model, many of the included studies would yield higher rCBV estimation errors compared to the optimized acquisition parameters [23, 25, 32, 34, 38]. In contrast, studies with optimized parameters would be expected to yield lower rCBV estimation errors and, interestingly, predominantly reported higher classification performance [26, 28, 30, 35]. For example, the study by Kickingereder et al. [28], predicted to yield the minimum rCBV estimation error, correctly classified 88% of tumors using rCBV 90^th^ percentile (accuracy range: 82–88% for all percentiles). Such an assessment of methodological quality is limited since it requires complete protocol and post-processing data which are not available for all studies and thus does not allow appropriate comparisons. However, it can be postulated that errors in estimating rCBV may influence the classification performance of DSC perfusion and this insight can highlight areas for optimization of the technique. In addition, when using a shorter TR, the T_1_ of blood is minimized allowing for more accurate measurements of rCBV which may better expose the subtle differences in perfusion between IDHm and IDHwt. Lastly, smaller slice thicknesses and slice gaps would increase the image resolution. This would magnify any existing differences in glioma vascular signatures between IDHm vs. IDHwt leading to a better discriminative power of DSC. However, this obviously comes at the expense of longer acquisition times.

### Perfusion metrics—novel methodologies

A wide range of DSC metrics were reported by the included studies (Tables 2 and 3). Although rCBV_mean_ was the most widely employed, it may not be the optimal metric for capturing the vascular signatures of gliomas. Indeed, Choi et al. [24] suggested that specific segments of the signal intensity time curve may exhibit a better predictive performance for IDH mutation status. More specifically, IDHwt demonstrated a steeper downslope of signal intensity in the initial parts of the curve and less steep upslope in the region of signal recovery. Such minute temporal discriminatory features may be lost when using aggregate metrics such as rCBV_mean_. A novel metric might be the percentage signal recovery ratio (PSR) which better characterizes the DSC signal intensity curve and was successfully employed for IDHm status prediction [25]. Similarly, M-enhanced analysis of the DSC signal intensity curve is also an exciting prospect [9]. The low number of studies employing such metrics or methodologies prevented us from conducting meta-analyses. However, these innovations warrant further research and external validation.

Studies employing histogram analysis of DSC perfusion maps reveal histogram features as potential predictors of IDHm status [8, 27, 29]. Despite the smaller sample sizes, our findings highlight the discriminative value of the extreme rCBV percentiles for IDH prediction. When distinguishing IDHm from IDHwt, the highest sensitivities were observed for rCBV 10^th^ (i.e., 92% [86, 96]), 75^th^ (i.e., 91% [80, 96]) and 90^th^ percentiles (i.e., 88% [62, 97]). Thus, the use of histogram features for predicting IDH status warrants further investigation.

### Radiogenomics and machine learning

Radiogenomics introduce further capabilities for non-invasive glioma genotyping. This is highlighted by a recent systematic review which reported a high diagnostic performance (i.e., pooled sensitivities > 90% and pooled specificities > 87%) for both the prediction of IDH and 1p19q status [13]. By applying advanced machine learning models to imaging techniques such as DSC perfusion, there is a potential for improved performance in genotype status prediction [8]. However, benefits may extend beyond glioma genotyping to precision treatment selection and patient surveillance through the identification of independent vascular profiles. Recent studies demonstrate this capability, with promising outcomes for metrics beyond rCBVmean [24–26, 28]. Such applications would further benefit from improved DSC acquisition protocols as optimized parameters associating with a higher discriminative performance may have synergistic effects with the machine learning models. This highlights the importance of this study, as we characterize acquisition parameter trends associated with a higher diagnostic performance in distinguishing IDHm from IDHwt.

### Limitations

Despite utilizing robust methodologies and performing stratified random-effects meta-analyses, our study has several limitations. Firstly, we did not prospectively register the study protocol in a systematic review database such as the International Prospective Register of Systematic Reviews (PROSPERO). Prior registration is considered to increase transparency and reduce the likelihood of bias. Also, assessment of the performance of DSC perfusion in grading gliomas posed inherent limitations due to the inclusion of studies prior the transition to the current 2021 WHO classification. Although we believe our results on glioma grading provide insights in the value of DSC perfusion in predicting perfusion phenotypes related to microvascular proliferation and necrosis, they are insufficient to assess grading in accordance with current criteria, which remains to be investigated. Furthermore, we did not include studies employing machine learning–enhanced DSC perfusion since their methodology would not allow the assessment of DSC in isolation. Similarly, we did not explore the role of other MR perfusion techniques such as DCE or arterial spin labeling which might be better suited for non-invasive glioma genotyping. Considerable inter-study heterogeneity was identified by our analysis, and this prevented us from providing generalizable cut-off values to distinguish IDHm from IDHwt, and IDHm with 1p19q codeletion and IDHm without 1pq19q codeletion. To explore sources of heterogeneity, we performed meta-regression which highlighted moderator variables associated with a higher discriminatory performance for meta-analyses including at least 4 studies. However, the number of studies in most meta-analyses and meta-regressions was small. The latter limits the generalizability of our findings and warrants further investigation. Similarly, limitations in the number of studies precluded a diagnostic accuracy meta-analysis for the prediction of 1p19q codeletion status in IDHm, which remains a relevant research question.

### Research directions

Our study indicates a promising diagnostic performance of DSC perfusion for the non-invasive prediction of IDH mutation and 1p19q codeletion status, and subsequently for glioma grading based on the current WHO classification. We propose that limitations in diagnostic performance may not reflect inherent inadequacies of DSC perfusion. Rather, this is potentially attributed to the heterogeneity in acquisition protocols and post-processing as well as to limitations of commonly used perfusion metrics which fail to capture the dynamic properties of the signal-intensity curve. Research on optimization DSC perfusion protocols and on alternative novel perfusion metrics beyond rCBV may open new horizons for the clinical applications of DSC. This may not be limited to pre-treatment subtyping of gliomas, but could extend to personalized treatment selection and post-treatment surveillance. Machine learning can further enhance such capabilities and, in addition, allow the incorporation of multiparametric data from complementary advanced techniques (e.g., MRS).

## Conclusions

DSC perfusion shows a great potential in predicting IDH mutation and 1p19q codeletion status in gliomas. Heterogeneity in acquisition and post-processing warrants further research prior its wide adoption; however, previously unexamined aspects of the technique offer new prospects for enhancing its capabilities in clinical neuro-oncology. Technical standardization and optimization of DSC perfusion remain fundamental in the era of machine learning enhanced radiogenomics.

## Supplementary information

Below is the link to the electronic supplementary material.

Supplementary file1 (XLSX 14 KB)

Supplementary file2 (DOCX 48 KB)

## Data Availability

Data will be published with the manuscript.

## Abbreviations

DSC: Dynamic susceptibility contrast (MR perfusion)
IDH: Isocitrate dehydrogenase
TE: Echo time
TR: Repetition time
rCBV: Relative cerebral blood volume
DCE: Dynamic contrast enhanced (MR perfusion)

## Code availability

Code is available from the corresponding author upon reasonable request.
