# Supplementary material for: The role of DSC MR perfusion in predicting IDH mutation and 1p19q codeletion status in gliomas: meta-analysis and technical considerations
Source: Neuroradiology. 2023 May 13;65(7):1111–26. doi: 10.1007/s00234-023-03154-5 (PMC10272274; doi:10.1007/s00234-023-03154-5)
Supplement: Supplementary file 2 — Supplementary file2 (DOCX 48 KB) [file 234_2023_3154_MOESM2_ESM.docx]

**Supplementary Material 2 for:**

**The role of DSC MR perfusion in predicting IDH mutation and 1p19q codeletion status in gliomas: meta-analysis and technical considerations**

Loizos Siakallis ^1,2*^, Constantin-Cristian Topriceanu ^1,2,4*^, Jasmina Panovska-Griffiths ^5,6^, Sotirios Bisdas ^2,3^

*Joint 1^st^ authors.

Author Affiliations:

1. University College London (UCL) Queen Square Institute of Neurology, London, UK
2. Lysholm Department of Neuroradiology, The National Hospital for Neurology and Neurosurgery, University College London Hospitals (UCLH) NHS Foundation Trust, London, UK
3. Department of Brain Repair & Rehabilitation, Queen Square Institute of Neurology, University College London, London, UK
4. UCL Institute of Cardiovascular Science, University College London, London
5. The Big Data Institute and the Pandemic Sciences Institute, Nuffield Department of Medicine, University of Oxford, Oxford, UK
6. The Queen’s College, University of Oxford, Oxford, UK

**Corresponding author:**

Loizos Siakallis

UCL Queen Square Institute of Neurology, Queen Square, London WC1N 3BG, United Kingdom.

Tel: 0044 7467159092

Email: loizos.siakallis.20@ucl.ac.uk

**Supplementary Table S1. Standardized mean difference meta-regression.**

| Moderator name | Moderator type |  | All WHO | | WHO III | WHO IV | |
| --- | --- | --- | --- | --- | --- | --- | --- |
| Moderator | Moderator |  | rCBVmean | rCBVmedian | rCBVmean | rCBVmean |  |
|  |  | Number of studies | 7 | 4 | 3 | 3 |  |
| Contrast dose | Continuous | β (95% CI) | -7.06 (-12.84, -1.27) | -5.36 (-10.33, -0.39) | N/A | N/A |  |
|  |  | p-value | **0.017** | **0.035** | N/A | N/A |  |
|  |  | Number of studies | 7 | 4 | 3 | N/A |  |
| TE | Continuous | β (95% CI) | 0.04 (-0.00, 0.08) | 0.05 (0.01, 0.09) | 0.09 (0.01, 0.17) | 0.07 (-0.01, 0.14) |  |
|  |  | p-value | 0.051 | **0.024** | **0.026** | 0.073 |  |
|  |  | Number of studies | 7 | 4 | 4 |  |  |
| TR | Continuous | β (95% CI) | 0.001  (0.00, 0.001) | 0.00 (-0.00, 0.00) | 0.00 (-0.00, 0.00) | 0.00 (-0.00, 0.00) |  |
|  |  | p-value | **0.044** | 0.301 | 0.451 | 0.993 |  |
|  |  | Number of studies | 7 | 4 | 4 |  |  |
| Slice thickness | Continuous | β (95% CI) | 0.18 (-0.07, 0.42) | 0.54 (0.04, 1.03) | 0.36 (-0.42, 1.13) | 0.36 (-0.41, 1.12) |  |
|  |  | p-value | 0.164 | **0.035** | 0.365 | 0.359 |  |
|  |  | Number of studies | 7 | 4 | 4 | 4 |  |
| Slice gap | Continuous | β (95% CI) | 0.08 (-0.47, 0.62) | N/A | N/A | N/A |  |
|  |  | p-value | 0.779 | N/A | N/A | N/A |  |
|  |  | Number of studies | 3 | 3 | 3 | 3 |  |
| Acquisition time | Continuous | β (95% CI) | -0.00 (-0.05, 0.05) | N/A | N/A | N/A |  |
|  |  | p-value | 0.983 | N/A | N/A | N/A |  |
|  |  | Number of studies | 4 | N/A | 1 | 1 |  |
| ROI selection | Categorical | Number of studies | 8 | 4 | 4 |  |  |
|  | Automated |  | ref | N/A | ref | ref |  |
|  | Manual hotspot | β (95% CI) | -0.27 (-1.46, 0.91) | -0.00 (-0.89, 0.89) | -0.39 (-2.87, 2.09) | -0.83 (-1.84, 0.17) |  |
|  |  | p-value | 0.652 | 0.9991 | 0.796 | 0.105 |  |
|  | Manual tumour parenchyma | β (95% CI) | -0.11 (-1.26, 1.04) | Ref | 0.23 (-1.90, 2.34) | 0.27 (-0.47, 1.01) |  |
|  |  | p-value | 0.850 |  | 0.835 | 0.468 |  |
| DSC Normalization  /Standardization | Categorical | Number of studies | 6 | 4 |  |  |  |
|  | Normalized |  | ref | N/A | ref | ref |  |
|  | Standardized | β (95% CI) | 0.28 (-0.87, 1.52) | N/A | 0.01 (-1.39, 1.41) | 0.10 (-1.24, 1.44) |  |
|  |  | p-value | 0.635 | N/A | 0.992 | 0.883 |  |
| AIF | Categorical | Number of studies | 5 | 1 | 3 | 3 |  |
|  | Automated |  | ref | N/A | N/A | N/A |  |
|  | Manual | β (95% CI) | -0.28 (-0.63, 0.07) | N/A | N/A | N/A |  |
|  |  | p-value | 0.121 | N/A | N/A | N/A |  |
| Leakage correction (includes contrast pre-loading) | Categorical | Number of studies | 6 | 4 | 3 |  |  |
|  | No |  | ref | ref | N/A | 3 |  |
|  | Yes | β (95% CI) | -0.64 (-1.02, -0.26) | 0.09 (-0.94, 1.12) | N/A | N/A |  |
|  |  | p-value | 0.964 | 0.862 | N/A | N/A |  |

Meta-regression has been performed only when the moderator was available in 4 or more studies.

AIF = arterial input function; β = beta regression coefficient; CBV = cerebral blood volume; CI = confidence interval; N/A = not applicable; ref = reference; ROI = region of interest; TE = time to echo; TR = repetition time, WHO = World Health Organization

**Supplementary Table S2. Diagnostic accuracy meta-regression.**

| Metric | rCBVmean All WHO grades | | | | | |
| --- | --- | --- | --- | --- | --- | --- |
| Moderator | Moderator type | Number of studies | Pooled Sensitivity per Subcategory (95% CI) or β (95% CI) | Pooled Specificity per Subcategory  (95% CI) or β (95% CI) | Bivariate Model  p-value for Sensitivity | Bivariate Model  p-value for FPR |
| Contrast dose | Continuous | 6 | 5.03 (-19.15, 29.21) | 1.97 (-12.57, 16.51) | 0.684 | 0.265 |
| TE | Continuous | 5 | -0.10 (-0.19, -0.02) | -0.6 (-0.13, 0.01) | **0.022** | 0.080 |
| TR | Continuous | 5 | 0.00 (-0.00, 0.01) | -0.00 (-0.00, 0.00) | 0.134 | 0.174 |
| Pulse-sequence | Gradient-echo Echo-planar | 1 | 52.00 (43.27, 60.61) | 90.80 (87.22, 93.45) | ref | ref |
|  | Gradient-recalled T_2_^*^ weighted echo-planar | 3 | 67.08 (48.39, 81.57) | 85.17 (75.98, 91.25) | 0.179 | 0.437 |
|  | MPRAGE | 1 | 88.46 (67.46, 96.59) | 75.00 (56.76, 87.27) | 0.390 | 0.317 |
| Slice thickness | Continuous | 5 | -0.15 (-1.27, 1.57) | -0.15 (-1.12, 0.82) | 0.834 | 0.760 |
| Slice gap | Continuous | 3 | -8.78 (-13.58, -3.97) | -4.89 (-8.65, -1.13) | **<0.0001** | **0.011** |
| Acquisition Time | Continuous | 3 | 0.03 (-0.01, 0.06) | 0.00 (-0.04, 0.05) | 0.107 | 0.869 |
| ROI selection | Automated | 1 | 52.00 (43.27, 60.61) | 90.80 (87.22, 93.45) | ref | ref |
|  | Manual Hotspot | 1 | 97.90 (87.89, 99.67) | 63.00 (48.66, 75.36) | **0.008** | **0.011** |
|  | Manual Enhancing Tumour | 4 | 73.96 (55.28, 86.71) | 82.66 (73.90, 88.92) | 0.281 | 0.165 |
| DSC Normalization  /Standardization | Normalized | 1 | 52.00 (43.27, 60.61) | 90.80 (87.22, 93.45) | ref | ref |
|  | Standardized | 4 | 86.55 (61.22, 96.33) | 79.50 (65.82, 88.64) | 0.293 | 0.271 |
| AIF | Automated | 2 | 71.48 (38.10, 91.08) | 86.52 (73.53, 93.68) | ref | ref |
|  | Manual | 2 | 56.69 (45.33, 67.39) | 85.29 (71.35, 93.10) | 0.694 | 0.990 |
| Leakage correction (including contrast pre-loading) | No | 1 | 52.00 (43.27, 60.61) | 90.80 (87.22, 93.45) | ref | ref |
|  | Yes | 3 | 76.08 (51.23, 90.60) | 84.50 (74.78, 90.93) | 0.372 | 0.391 |

DSC = dynamic susceptibility contrast; FPR = false positive rate; MPRAGE = Magnetization Prepared RApid Gradient Echo. Other abbreviations as in **Supplementary Table S1**.
